# Application of Intelligent Modeling Method to Optimize the Multiple Quality Characteristics of the Injection Molding Process of Automobile Lock Parts

**DOI:** 10.3390/polym13152515

**Published:** 2021-07-30

**Authors:** Wei-Tai Huang, Chia-Lun Tsai, Wen-Hsien Ho, Jyh-Horng Chou

**Affiliations:** 1 Department of Mechanical Engineering, National Pingtung University of Science and Technology, Pingtung 912, Taiwan; weitai@g4e.npust.edu.tw (W.-T.H.); jack5920658@hotmail.com (C.-L.T.); 2 Department of Medical Research, Kaohsiung Medical University Hospital, Kaohsiung 807, Taiwan; 3 Department of Healthcare Administration and Medical Informatics, Kaohsiung Medical University, Kaohsiung 807, Taiwan; 4 Department of Electrical Engineering, National Kaohsiung University of Science and Technology, Kaohsiung 807, Taiwan; 5 Department of Mechanical Engineering, National Chung Hsing University, Taichung 402, Taiwan

**Keywords:** injection molding, Taguchi methods, fuzzy theory, warpage, shrinkage, conformal cooling, multiple performance characteristic index (MPCI)

## Abstract

This study focuses on applying intelligent modeling methods to different injection molding process parameters, to analyze the influence of temperature distribution and warpage on the actual development of auto locks. It explores the auto locks using computer-aided engineering (CAE) simulation performance analysis and the optimization of process parameters by combining multiple quality characteristics (warpage and average temperature). In this experimental design, combinations were explored for each single objective optimization process parameter, using the Taguchi robust design process, with the *L*_18_ (2^1^ × 3^7^) orthogonal table. The control factors were injection time, material temperature, mold temperature, injection pressure, packing pressure, packing time, cooling liquid, and cooling temperature. The warpage and temperature distribution were analysed as performance indices. Then, signal-to-noise ratios (S/N ratios) were calculated. Gray correlation analysis, with normalization of the S/N ratio, was used to obtain the gray correlation coefficient, which was substituted into the fuzzy theory to obtain the multiple performance characteristic index. The maximum multiple performance characteristic index was used to find multiple quality characteristic-optimized process parameters. The optimal injection molding process parameters with single objective are a warpage of 0.783 mm and an average temperature of 235.23 °C. The optimal parameters with multi-objective are a warpage of 0.753 mm and an average temperature of 238.71 °C. The optimal parameters were then used to explore the different cooling designs (original cooling, square cooling, and conformal cooling), considering the effect of the plastics temperature distribution and warpage. The results showed that, based on the design of the different cooling systems, conformal cooling obtained an optimal warpage of 0.661 mm and a temperature of 237.62 °C. Furthermore, the conformal cooling system is smaller than the original cooling system; it reduces the warpage by 12.2%, and the average temperature by 0.46%.

## 1. Introduction

With the development of the plastics industry, the injection molding process has become the most widely used technology for molding plastics, and the majority of plastic products are manufactured in this way. The injection molding process has various advantages, including excellent dimensional precision and stability, good surface accuracy, low cost, and ease of complex shape formation, thus making it a highly productive processing technique. The quality of injection molded products is mainly affected by the selection of the process parameters, such as injection time, injection pressure, plastic temperature, mold temperature, holding pressure, and holding pressure time, which will afford different qualities under different settings. Therefore, the selection and setting of the process parameters are important factors in injection molding. At present, injection molding technology is being utilized for the production of high-tech products, automotive parts, and household products [1]. The mold is designed to match the gates and cooling circuits, according to the complexity of the product structure during the injection molding process, considering the diversification of the product applications, the trend towards meeting diversified demands, and under the influence of functionality. In the actual mold-opening production, the process parameters are often determined by trial and error, or according to rules that are formulated by experienced experts; this makes quality improvement a challenging task [2]. Therefore, this experiment will use intelligent modeling for the single- and multi-objective optimization of the quality characteristics of the injection molding process parameters.

The simulation technologies of computer-aided design (CAD) and computer-aided engineering (CAE) are used to help developers analyze and predict problems and their causes in each part of the injection molding process. The simulation helps decrease the number of actual mold trials, reduce the cost and time spent, and improve the quality of the mold. In this study, CAE software is used for the mold flow analysis. The software mainly utilizes the finite element method (FEM) to simulate plastic in the mold cavity at various stages of injection molding, which can be used as a reference for setting the injection molding parameters and mold design, to facilitate rapid product development as well as to reduce product and mold development costs. Wang et al. [2] presented a numerical dynamic injection molding technology (DIMT), which is based on the finite element (FE) method. In the warpage optimization work, three kinds of structures, with different molding materials, are selected for comparison. The final warpage of each product is efficiently minimized by using a Gaussian process-based sequential optimization method. Jong et al. [3] used CAE analysis data to train the BPNN. The Taguchi orthogonal method is used to optimize the hyperparameters in the neural networks, to construct a neural network that can predict CAE analysis results. Studies show that the prediction of the maximum injection pressure and the maximum cooling time is pretty good. A study by Huang et al. [4] applied both computer-aided engineering (CAE) simulation and experimental methods to investigate the fiber feature in a co-injection system. The fiber orientation distributions, and their influence on the tensile properties for the single-shot and co-injection molding, have been discovered. The results show that, based on the 60:40 skin/core ratio and the same materials, the tensile properties of the co-injection system, including the tensile stress and modulus, are a little weaker than those of the single-shot system. From these CAE analysis results, it can be seen that both the improvement in the quality of injection molding and the improvement in the performance of injection molding materials have a significant improvement effect.

At present, plastic injection molding is widely used in the manufacturing of automotive components, including bumpers, lights, dashboards, and connector parts. Among them, auto lock parts have extremely high-precision requirements. Their structure is complex, and the finished products are easily subject to warpage deformation, volume shrinkage, and weld line, making the selection and setting of the control process parameters even more important. Problems with the parts, related to warpage, can arise, which could be solved by adjusted process parameters to meet the part requirements [5]. Polymer parts suffer from shrinkage and warpage during the injection molding process, and are induced by thermal and pressure changes that are achieved over them [2]. As a result, the final dimensions of the injected components are affected by material shrinkage during the process, caused by the filling orientation, packing conditions, and cooling parameters [6,7]. For the selection of the warpage deflection of the injection molded products and related parameters of the injection molding process, the following related literature introductions have been sorted out. Rosaa et al. [8] believed that the experimental design should be widely used in order to optimize the molding parameters, to improve the quality characteristics of the product. Conventional experimental design methods are often complex and often fail to achieve the desired results. Moreover, these methods require a large number of experiments when the number of molding parameters is increased. Therefore, the use of the Taguchi orthogonal method for selecting experimental data may reduce the number of experiments. With respect to the defects produced in injection molding, Marinset et al. [9] proposed the use of the Taguchi method and the analysis of variance (ANOVA), to evaluate the effect of injection molding parameters on bending and shrinkage. They used two different plastics and mold temperatures, while the holding pressure, holding time, plastic temperature, cooling time, cooling water speed, and injection speed, were kept constant. The experimental results showed that the control factors of warpage and bending defects have the greatest influence on the holding time and holding pressure. Furthermore, to reduce the molding cycle time, Dimla et al. [10] performed a finite element analysis to find the optimal cooling circuit design for injection molding, and found that the cooling circuit and gate had the greatest effect on the injection cycle time. They performed further optimization, and, finally, using simulation analysis, they found that the molding cycle time was reduced and the surface condition of the product was significantly improved. This study will refer to the above-mentioned studies to select relevant injection molding control parameters and levels.

The following related studies are in the related research of different cooling water systems, and the development and application of conformal cooling. Park and Pham [11] proposed the use of the CAE software to analyze the molding temperature distribution of their products, designed a conformal cooling circuit system using the temperature distribution and product shape, compared the traditional cooling circuit with their design, and verified, using CAE, that this new method can make the temperature distribution more uniform. Ahn [12] discussed the manufacturing methods of various conformal cooling circuit molds, briefly described the development of a conformal cooling circuit design technology, introduced various production methods of conformal cooling circuits, and analyzed their respective heat transfer methods. Agazzi et al. [13] proposed a design method for cooling circuits. Through analysis and observation of the cooling temperature of the plastic inside the product and the cooling temperature distribution of mold, they designed a cooling circuit based on the temperature distribution. The results proved that the design method was effective. Juan et al. [14] proposed the use of an analysis software for the design and performance verification of the cooling circuit system of injection molding. For two thin-walled products, the automatically and manually designed cooling circuits were compared by a software, and the manually designed cooling circuit was found to greatly improve the warpage, according to the product shape. Wang et al. [15] explored the cooling circuit design of a complex automotive part interior. First, finite element analysis was used to obtain the mold temperature distribution, and then, the improved cooling circuit design was analyzed and verified. The method can make the mold temperature uniform, and improve the surface accuracy of the plastic part. From these related studies, it can be found that the use of conformal cooling is much higher than the traditional cooling water system, in terms of heat conduction simulation analysis and actual process application measurements of the cooling benefits. Therefore, this study will first optimize the process, and then use the optimized results to compare different forms of conformal cooling. The aim is to study the impact of the cooling benefits of automobile lock parts on the target quality characteristics.

The following related studies are part of using the intelligent modeling method optimization of injection molding performance. In a related study, Ozcelik and Erzurumlu [16] applied CAE as a research tool for the minimum warpage values in injection molding processes, designed experiments using DOE and Taguchi orthogonal tables, and used ANN and GA methods in combination to find the optimal warpage values. Ko-Ta Chiang and Fu-Ping Chiang [17] used a fuzzy gray-order method to explore the optimization of the process parameters of cell phone cases, and selected the following four control factors: mold temperature, plastic temperature, injection pressure, and filling time. Through finite element analysis, they found that the main factors that affected volume shrinkage and temperature distribution were the mold temperature and holding pressure; the volume shrinkage was reduced from 0.012 to 0.007 mm, and the temperature distribution decreased from 8.510 °C to 7.345 °C. Cheng et al. [18] discussed the optimization of mold performance using fuzzy theory analysis, and validated the fuzzy theory method based on the creation of three molds. The seriousness of the defect was categorized as very slight, slight, medium, severe, and very severe, according to the triangular membership function. Six criteria, including short shot, weld line, sink mark, volume shrinkage, air trap, and warpage, were discussed. A study on the optimization of mold schemes proved this method to be effective and feasible. However, these related studies all use intelligent modeling methods to optimize a single goal. In the current industrial environment, where production efficiency is emphasized, the optimization of multiple goals at the same time is required, in order to improve the product performance greatly. The intelligent modeling method that was used in this study integrates multiple optimization algorithms, which can optimize the quality characteristics of a single goal and multiple goals simultaneously.

This study examines a set of auto lock parts in actual production. The warpage deformation of parts during the manufacturing process has made the parts unfit for assembly. In actual production, important parameters have to be found through trial-and-error, to determine whether the selected parameter settings need to be changed after observing the product quality, which can greatly increase the cost and time. Herein, CAE software is used, with intelligent modeling methods, to solve this problem. Due to the uneven distribution of the thickness of the structure, the uneven temperature distribution can result in shape and size deformation of the product, thus affecting the quality of the subsequent assembly, in which case the process parameters are changed in order to reduce the warpage deformation. In order to confirm the accuracy of CAE software analysis, we performed calibration before the start of the experiment. The actual car lock parts in actual production are used for calibration and comparison analysis with the original process parameters, which has ensured the correctness and accuracy of the experimental results; it is not because there are only simulated data experiments. In addition, the intelligent modeling method that was used in this experiment is also the first, in the related research, to combine the results of Taguchi’s experimental data with gray correlation and fuzzy theory analysis, to conduct research that can simultaneously optimize a single objective and multiple objectives. First, the CAE mold flow analysis software is used with the Taguchi robust process design method, to find the combination of each single quality optimization parameter, and the warpage deformation and average temperature are discussed separately. Combining the results of Taguchi’s experimental data with gray correlation and fuzzy theory analysis, the optimal combination of a multi-objective quality process was found [19], and the warpage deformation and average temperature were then compared with those of the original process. Finally, comparison and analysis were performed for the parameter combination of the multi-objective quality optimization process in different system designs of cooling circuits, namely, original cooling, square cooling, and conformal cooling.

The Taguchi method is a robust design method that uses the concept of statistical experimental design. Orthogonal array (OA) can analyze a large number of design variables through some experiments. Since OA is a fractional factorial matrix, it ensures a balanced comparison of the level of any factor or the interaction of the factors. Using OA to collect appropriate data, and applying intelligent modeling methods to optimize the multiple quality characteristics of the automotive lock injection molding process, can reduce development and manufacturing costs. In comparison with this experimental method, Wang et al. [15] studied automotive parts, similar to the subject of this study, and the parts were three-dimensional complex-shaped automotive interior parts. The injection mold on the heating/cooling system design of the part was studied. In the experimental design, the full factorial experiment is used without any experimental method, and only two control factors are used in the experimental control factor. The mold heating time has four levels, and the mold cooling time has five levels. There are only two control factors, but the number of experiments needs to be as many as 20 (4 × 5 = 20) times. It can be observed that if the current industry development trend needs more factors, and levels must be controlled at the same time, the number of experiments will be greatly increased. As a result, the experiment cost and time increased significantly, which could not effectively improve the efficiency and immediately solve the practical problems in industrial production. In this article, for each quality feature of the eight control factors, if we do not use Taguchi’s method to collect the experimental data, the number of full-factor experiments is 4374 (2 × 3^7^ = 4374). However, *L*_18_ (2^1^ × 3^7^) only has 18 experiments to collect appropriate data for each quality feature. While optimizing the process parameters of the combination of fuzzy logic and multiple performance characteristic index (MPCI) in the injection molding process of automobile lock parts, all the quality characteristics in the injection molding process can be considered simultaneously. The current industry practices are measured by engineers’ field experience, which introduces a lot of uncertainty. The fuzzy logic program used in MPCI reduces the uncertainty that is caused by humans, and does not require complicated mathematical calculations. The system simulation in this study applied fuzzy logic in MPCI. Compared with conventional methods, the output can better meet the requirements of technical engineers and customers. Practical applications can further encourage the transfer of fuzzy logic-based technology from academia to industry.

## 2. Experiment Configuration

### 2.1. Construction of Auto Lock Spare Parts

**Figure 1:**
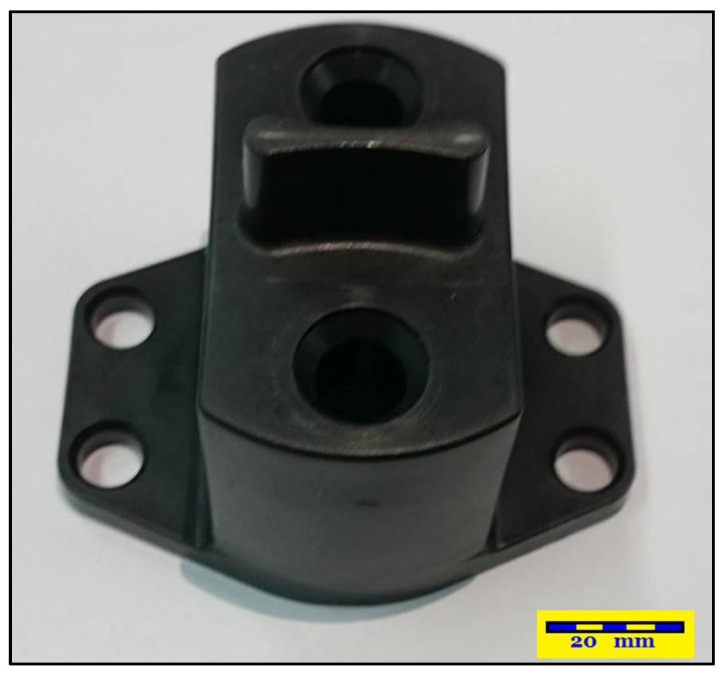
Actual image of an auto lock part.

**Figure 2:**
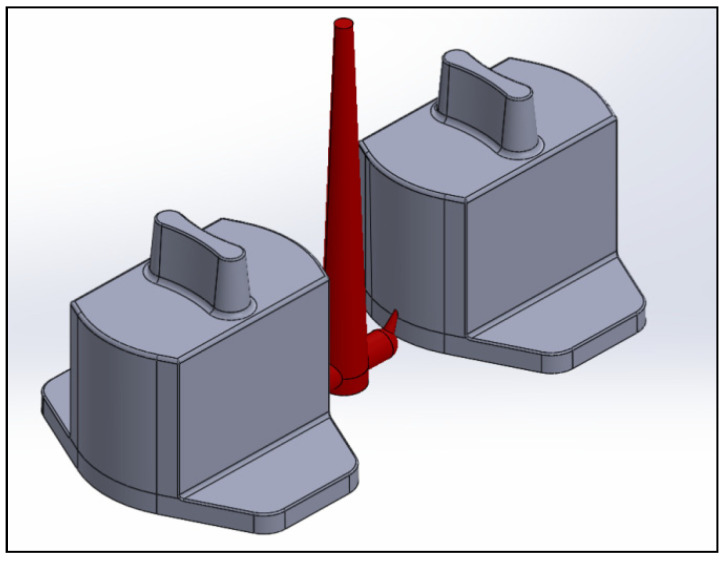
Mold configuration.

**Figure 3:**
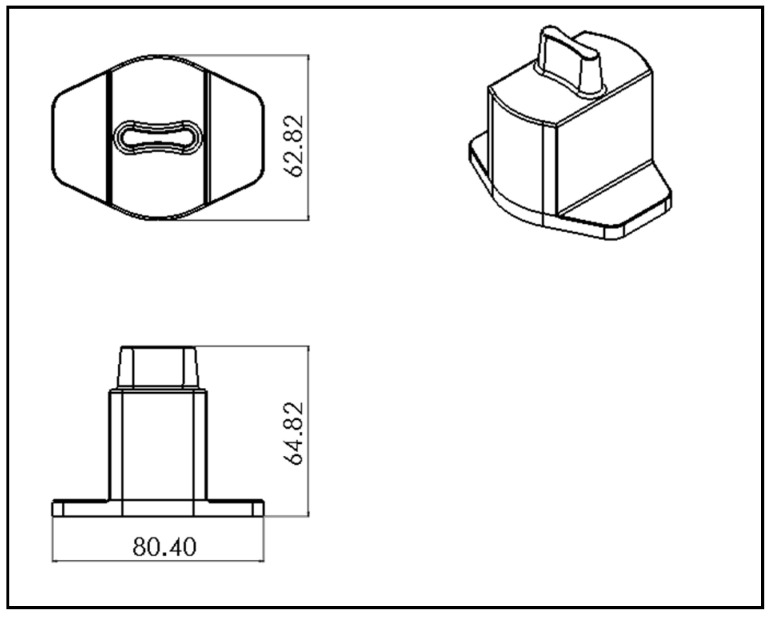
Dimensions of parts (dimensions are in mm).

**Figure 4:**
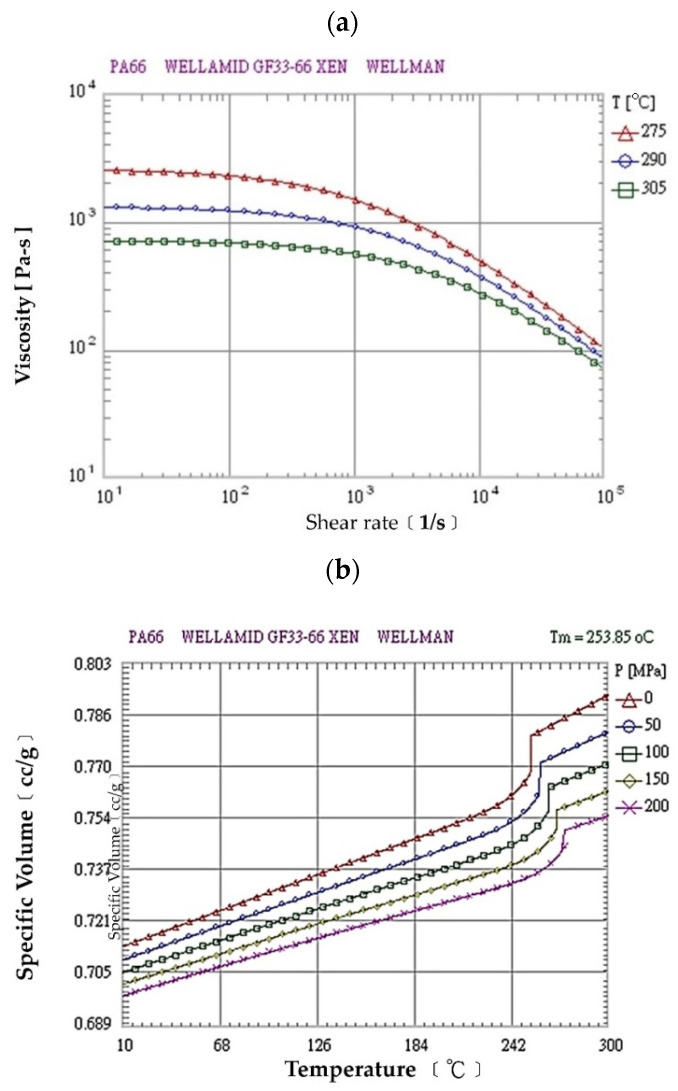
Material properties of PA66 (6212GC): (**a**) viscosity vs. shear rate; (**b**) specific volume vs. temperature.

The actual shapes of the auto lock parts that were studied in this study are shown in Figure 1. According to the original design for the configuration and drawing of the double-cavity mold (Figure 2), the dimensions of the parts are as follows: the length is 80.40 mm, the width is 62.82 mm, and the height is 64.82 mm. Figure 3 shows the original design of the cooling circuit, which has a circumferential structure, with a diameter of 6 mm. Polyamide 66 (PA66) is a thermoplastic polymer that has been widely used in automotive-related component materials, due to its excellent mechanical and thermal resistance, good barrier properties, and recyclability [20]. The selected plastic material is engineering plastic nylon (polyamide, PA66), a composite polymer of 6212GC that is produced by Nan Ya Plastics Corporation, containing 33% glass fiber. This material is especially suitable for parts that require high rigidity and high toughness. For example, the automobile lock parts in this experiment need to be repeatedly used in the car’s high-temperature and low-temperature environments for a long time, so the material itself must have high rigidity and high toughness. Table 1 shows the basic properties of the materials. The viscosity and specific volume properties of the PA66 (6212GC) are shown in Figure 4a,b, respectively. As the CAE software used in this study is Moldex3D, there is only general PA66 material characteristic data in this built-in material library. There are no material characteristic data of this part material PA66 (6212GC). To make the experimental results more accurate, the source of the material property data in Table 1, and Figure 4a,b, are used to commission the Moldex3D Material Science Research Center to carry out the material property data before starting the experiment. The center has passed ISO 17025 international certification. The dynamic viscosity is measured using a cone-and-plate/parallel-plate viscometer. The specific heat capacity is measured using a differential scanning calorimeter.

The CAE software that was used in this study was Moldex3D, with the Moldex3D/Solid analysis module being the main analysis tool, followed by the Moldex3D-Mesh module. First, the 3D drawing software was used to construct the model, and then, the completed model, cooling circuit, sprue channel, and mold were assembled and imported into the software for setting. Thereafter, the Moldex3D/Mesh software was used to construct the solid meshes for the cooling circuits and products, thereby establishing the tetra solid mesh, which has approximately 1.25 million meshes and 1.2 million nodes in this study. Finally, the injection molding parameters were set and the analysis results were interpreted.

### 2.2. Simulation Analysis of Original Injection Process Parameters and Comparison with Actual Plastic Parts

**Figure 5:**
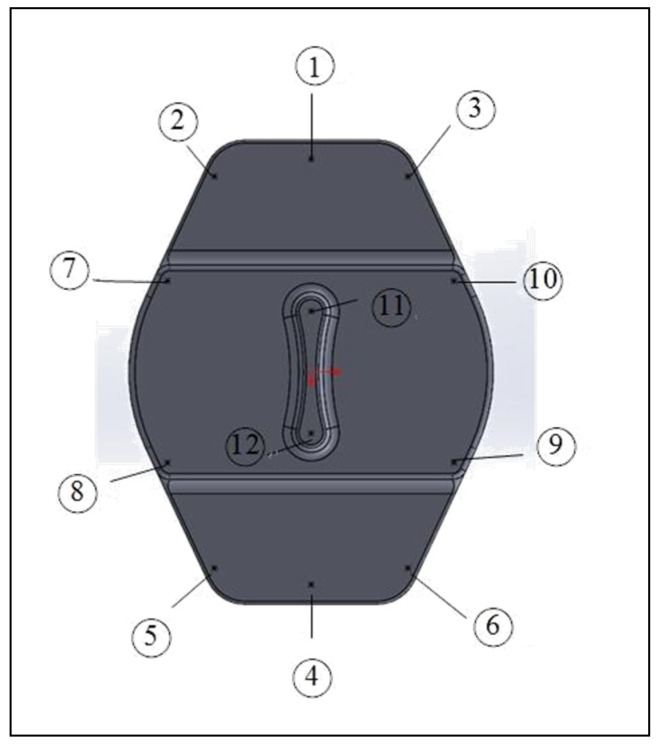
Schematic of the locations of the measurement points for auto lock part.

**Figure 6:**
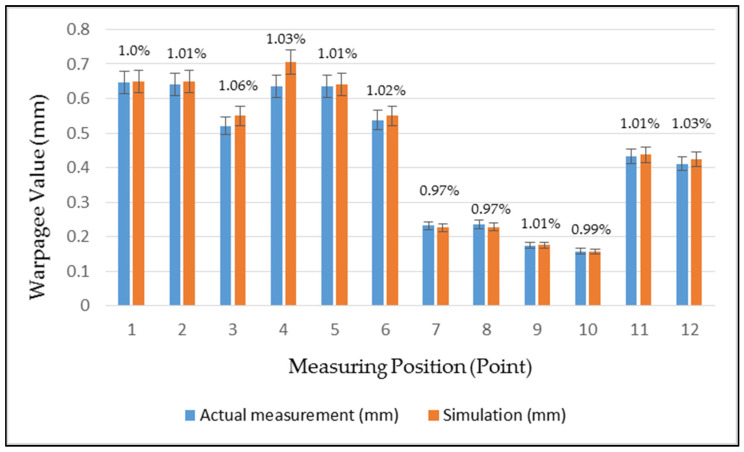
Comparison of actual and analog warpage value measurement points.

To understand the existing condition of the auto lock parts, an analysis was first performed by setting the actual process parameters, given by the original manufacturer, as the original process parameters, and the results were analyzed through CAE software simulation. To make the simulation analysis consistent with the actual situation, the warpage measurement of the existing product was first performed with a 3D measuring instrument (Tesa Micro-Hite 3D 4.5.4, with a measurement accuracy of 0.001 mm). Mutual verification with CAE software simulation results was performed. Twelve points were selected for the measurement of the parts, each point was measured three times and the average value was taken, and the same points were selected for the experiment, as shown in Figure 5. The comparison between the actual measured warpage values in the Z-direction and the analysis result of the warpage deformation in the *Z*-axis direction, is shown in Table 2. Figure 6 shows the trend comparison chart for verifying that the simulation results are consistent with the actual production conditions. There will be different changes in the geometry design of the injection molded parts, which will cause different parts in each area of the part to have different part wall thickness changes, which will also cause the difference in the dimensional stability of the part area. From the comparison result of Figure 6, it can be found that points 1–6 have larger warpage values because of the thin wall thickness of the parts in this area. At points 7–10, because the wall thickness of the parts in this area is thicker, there are relatively small warpage values. Related research results show that PA66 is very sensitive to different cooling conditions. The material has different crystallization changes during the cooling process, due to the different cooling conditions inside and outside the material, which affects the dimensional stability of the part [21]. Therefore, this study was also carried out after optimizing the injection parameters—comparison and analysis of different cooling systems. 

### 2.3. Overall Experiment Flow

**Figure 7:**
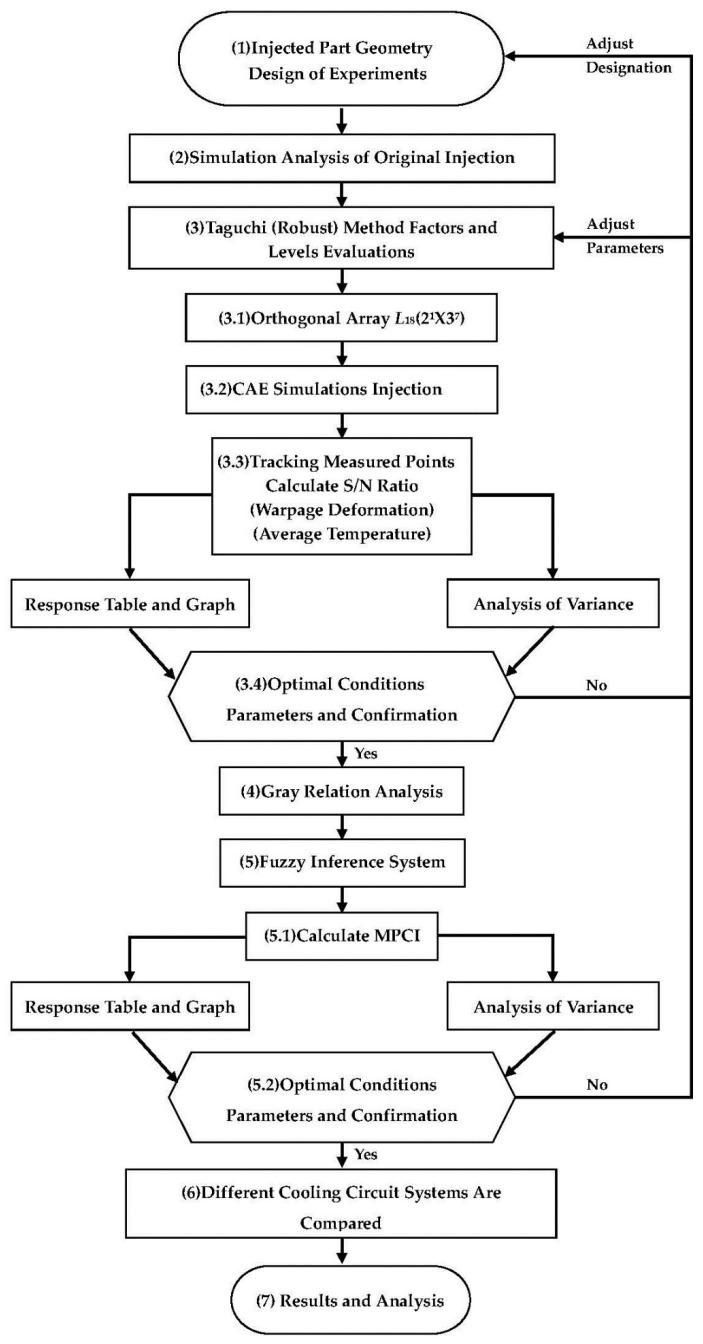
Overall experiment flow chart.

The experimental framework of this study can be divided into three main parts. The overall experimental flow chart is shown in Figure 7. First is the experimental design part in (3) of Figure 7; the Taguchi robust process design method is applied for the single-objective optimization design for the warpage and average temperature of an auto lock part. Second is the experimental design part in (4) and (5) of Figure 7, in which gray correlation and the fuzzy theory are used, respectively. Then, the first and second steps are used to find the optimal parameter combination of multiple quality characteristics of warpage and average temperature. Third is the experimental design part in (6) of Figure 7, which refers to the related conformal cooling research. This experiment will be the optimal parameter combination of multiple quality characteristics in different cooling circuit systems, such as original cooling, square cooling, and conformal cooling, and their effects on warpage and average temperature are also compared [22,23].

### 2.4. Taguchi Robust Design Process

In this study, the *L*_18_ (2^1^ × 3^7^) orthogonal table is used to perform the experiments. The arrangement of the combined parameters on the orthogonal table is used to perform the simulation analysis of the mold flow. Through the signal-to-noise (S/N) value, the best parameters for the injection molding of automobile lock parts can be obtained. The S/N value that was obtained through the orthogonal table planning experiment, can be used to find the relevant data of ANOVA, perform the confirmation experiment, and observe the contribution of each factor. As shown in Table 3 (the multi-objective analysis flow of the Taguchi robust process design method), the warpage and average temperature of the auto lock part are first separately used for single-objective optimization; the control factors are injection time, material temperature, mold temperature, injection pressure, packing pressure, packing time, cooling liquid, and cooling temperature. The quality characteristics to be optimized in this study are the total warpage and average temperature of the auto lock parts, with the expectation that the warpage and shrinkage are as small as possible.

The measured values of the two target total warpage and average temperature values are measured after the mold parting surface is opened, after the entire injection molding process is completed, and the molded product is demolded, to measure these two target simulation values. In the CAE simulation environment, there will be no temperature and pressure difference, due to the long time contact with the ambient temperature after the molded product is demolded, and the accuracy of the experimental value can be ensured. Generally, PA66 is easily affected by moisture absorption, which significantly impacts its dimensional stability and mechanical properties. However, the PA66 (6212GC) composite polymer containing 33% glass fiber was used in this experiment. Generally, in an environment of 20 °C and 50% relative humidity, the saturated moisture content of PA66 is 2.3~2.8%, and the saturated moisture content of PA66 (6212GC) is 1.4~1.7%. In an environment of 20 °C and 100% relative humidity, the saturated moisture is 8.0~10.0%, and the saturated moisture of PA66 (6212GC) is 5.0~6.0%. As the PA66 (6212GC) material that was used in this experiment is added with glass fiber reinforced material, the dimensional stability of the molded product after moisture absorption is excellent. Therefore, the quality is defined as a static smaller-the-better (STB) characteristic. In this study, the parameters used are as follows: oil and water were selected as the cooling liquid, the packing time was in the 1–2 s range, the cooling temperature was in the 15–45 °C range, the packing pressure was in the 190–210 MPa range, the mold temperature was between 20 °C and 60 °C, the injection time was from 1 to 2 s, the material temperature was between 255 °C and 275 °C, and the injection pressure was in the 110–150 MPa range. Then, the warpage and average temperature conditions were further observed, in order to obtain the manufacturing process parameters of auto lock parts with the optimal design for each single objective. Taguchi methods are the most widely applied robust design methods in the planning of process parameters, because they reveal the effects of various combinations of parameters on a relevant single quality characteristic [24,25,26,27]. The combinations of parameters for the experimental injection molded sample were assessed with the Taguchi *L*_18_ (2^1^ × 3^7^) OA, using the warpage and average temperature as single quality characteristics. Small values of these characteristics are more favorable. Therefore, the optimization of the injection molded parameters was considered as a static problem with smaller-the-better S/N ratios ($S/N_{STB})$, which were expressed as Equation (1).

(1) $$S/N_{STB}=-10log_{10}\left[\frac{1}{n}\overset{n}{\underset{i=0}{\sum }}y_{i}^{2}\right]$$

where,

*n*: instances observed in each experimental combination;
$y_{i}$: the *i*-th datum in the experimental combination.

### 2.5. Fuzzy Theory Analysis

This study is mainly to solve the optimization of multiple quality characteristics. When only the objective function and conditional constraints are known, it is necessary to seek better results through mathematical programming methods. The gray correlation analysis method can convert multiple quality characteristics into a single gray correlation value. By comparing the gray correlation value, the response size of each quality characteristic value is arranged, so the best combination of factors can be selected. However, the gray correlation analysis assumes that the quality characteristics are independent of each other. Once there is a correlation between the quality characteristics, the gray correlation analysis cannot be resolved. However, to make the multi-objective quality optimization effect better, the design adopts the fuzzy theory method, and the basic spirit accepts the fact that fuzzy phenomenon exists. The application focuses on the experience of engineers and the characteristics of the problem mastery.

Grey relational analysis (GRA) provides a powerful method for solving multi-objective problems, and investigating the relationship between multi-factor and multi-variable feature optimization problems. GRA not only leaves the importance of multiple factors in a relatively complex unknown system to be explored, but also provides method results to determine which specific factors dominate the final quality. This method can usually be used to analyze the degree of influence of various factors on the results. It can also be used to solve comprehensive evaluation problems that change with time. The core is establishing a mother sequence that changes with time, according to certain rules and dividing each evaluation object over time. As a sub-sequence, find the correlation between each sub-sequence and the parent sequence, and draw conclusions based on the correlation. Since the two objectives of this experiment (total warpage and average temperature) are different physical quantities, it is impossible to compare their correlations and differences directly. It is necessary to use GRA to convert the S/N value of a total of 36 groups from 18 groups for each of the 2 targets, into a value between 0 and 1 to be consistent and input into the subsequent fuzzy inference system for the MPCI calculation.

The fuzzy theory is mainly a method of quantifying fuzzy concepts. The fuzzy theory does not advocate the use of complicated mathematical analysis and models to solve problems. It extends traditional mathematics from binary logic to continuous multi-value, and uses the membership function to describe a concept. The membership function is the basic concept of the fuzzy theory, which can be used to describe the properties of fuzzy sets. The fuzzy set can be quantified through the attribute function, and only accurate mathematical methods can be used to analyze and process the fuzzy information. The value ranges from zero to one, and is a function of the degree of belonging of the element. The basic structure of a complete fuzzy logic control system consists of five main parts, including fuzzification, fuzzy inference, data base, rule base, and defuzzification. The fuzzy inference system converts the input signal of a clear value into a fuzzy value, and the control rules are established by the user’s operating experience and knowledge. The fuzzy output value is generated through inference calculations. Finally, the fuzzy output value will be defuzzified. The system is controlled and optimized. The fuzzy inference system mainly includes definition variables, fuzzification, knowledge base, fuzzy inference, and defuzzification.

Based on the experimental results of the *L*_18_ (2^1^ × 3^7^) orthogonal table, the S/N ratios for a single quality characteristic, such as warpage deformation and average temperature, were first calculated and then normalized using gray correlation generation, as shown in Equation (2); the normalized value was between zero and one. The normalized data were then analyzed by gray correlation, to calculate the gray correlation coefficient, as shown in Equation (3) [28]. The fuzzy inference rules were developed to represent the changes in the input and output after the gray correlation coefficient was calculated and substituted into the fuzzy inference system. This study adopted the triangular membership functions for both the input and output variables, for which the following three levels are defined: S, M, and L. The output variables can be subdivided into seven levels, which are very small (VS), small (S), middle (M), large (L), and very large (VL); thus, a total of nine fuzzy inference rules can be defined, as shown in Table 4 [29]. The Matlab fuzzy tool was used to set two input parameters and one output parameter for fuzzy rule substitution. Finally, multiple quality characteristics were converted into a single measurement index (MPCI, multiple performance characteristics index), and the MPCI values were compared to select the optimal parameter combination of multiple quality characteristics.

(2) $$x_{i}^{*}\left(k\right)=\frac{x_{i}^{\left(0\right)}\left(k\right)-min_{\;all\;i}\;\left[\;x_{\;i}^{\left(0\right)}\left(k\right)\right]}{\;max_{\;all\;i}\left[x_{\;i}^{\left(0\right)}\left(k\right)\right]-min_{\;all\;i}\;\left[x_{i}^{\left(0\right)}\left(k\right)\right]}$$

where $x_{\;i}^{\;*}\left(k\right)$ is the generated value after gray correlation, $min_{\;all\;i}\;\left[x_{\;i}^{\left(0\right)}\left(\;k\right)\;\right]$ and $\;min_{\;all\;i}\;\left[x_{\;i}^{\left(0\right)}\left(k\right)\right]$ are the maximum and minimum values in the $x_{\;i}^{\;*}\left(k\right)$ sequence, respectively, and OB is the target value selected from $x_{\;i}^{\;*}\left(k\right)$.

(3) $$\gamma \left(x_{i}\left(k\right)\;,\;x_{j}\left(k\right)\right)=\frac{\Delta_{min}+\zeta \;\Delta_{\;max}}{\;\Delta_{\;0\;i}\left(k\right)+\zeta \;\Delta_{\;max}}$$

where $\gamma \left(x_{i}\left(k\right)\;,\;x_{j}\left(k\right)\right)$ is the gray correlation coefficient, $\Delta_{min}$ is the minimum difference, $\Delta_{max}$ is the maximum difference, $\Delta_{0\;i}\left(k\right)$ represents the series difference between the values of the parent series $x_{0}\left(k\right)$ and the child series $x_{i}\left(k\right)$ at corresponding positions, and *ζ* is the coefficient of discrimination, which generally takes a value of 0.5. The main function of the identification coefficient ζ is to compare the reference value and the object to be tested, and its value range is [0, 1]. The identification coefficient is usually 0.5. If it is to aggravate the difference in the results, the value can be adjusted appropriately according to the actual situation. The change in this data will only affect the relative value, and will not change the analysis result.

## 3. Experiment Results

### 3.1. Optimization of Process Parameters for Warpage Deflection as a Single Quality Characteristic

**Figure 8:**
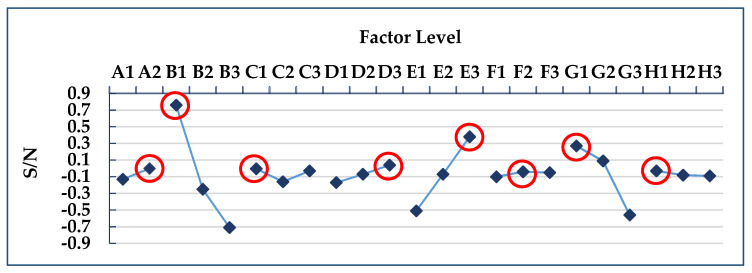
S/N factor response graph of warpage deformation.

The *L*_18_ (2^1^ × 3^7^) orthogonal table was used for the experimental simulation of 18 groups of process parameter combinations. This part is the experimental part in (3.1) and (3.2) of Figure 7. The warpage deformation of each group was simulated, and the S/N value of each group was calculated, respectively; the results are shown in Table 5. Table 6 shows the S/N factor response table, and Figure 8 shows the S/N factor response graph. This part is the experimental calculation result part in (3.3) of Figure 7. The degree of impact of each factor on the warpage deflection can be known from the factor response table and graph, which are in the following order: cooling liquid, holding pressure, cooling temperature, holding time, mold temperature, filling time, plastic temperature, and injection pressure. The optimized process parameters are A2B1C1D3E3F2G1H1, where A2 is the cooling liquid (oil), B1 is the holding pressure (190 MPa), C1 is the cooling temperature (15 °C), D3 is the holding time (2 s), E3 is the mold temperature (60 °C), F2 is the filling time (1.5 s), G1 is the plastic temperature (255 °C), and H1 is the injection pressure (110 MPa). However, this process parameter combination is not found in the *L*_18_ (2^1^ × 3^7^) orthogonal table, suggesting that a confirmation experiment must be conducted in order to compare with the group comprising the lowest warpage deformation in the *L*_18_ (2^1^ × 3^7^) orthogonal table, as shown in Table 7. The 10th group has the smallest warpage deformation among the 18 groups, with a warpage deformation of 0.807 mm, while the warpage deformation of the optimized parameter combination is 0.783 mm. Therefore, A2B1C1D3E3F2G1H1 can be concluded to be the optimized process parameter combination, with regard to warpage deformation. This part is the experimental result part in (3.4) of Figure 7.

In terms of ANOVA, as shown in Table 8, it can be understood, from the degree of contribution, that factor B, the holding pressure, will directly cause shrinkage changes within the product. Thus, the holding pressure is the parameter that has the greatest effect on the results of warpage deformation among the eight factors, followed by factor E, the mold temperature; factor G, the plastic temperature; factor D, the holding time; factor C, the cooling temperature; factor A, the cooling liquid; and factor F, the filling time. Factor H, the injection pressure, has the smallest contribution, indicating that Factor H has the smallest effect on the warpage deformation results. In the ANOVA analysis, degree of freedom (DOF) refers to the number of independent or freely variable data (levels) for each factor that is controlled by the Taguchi method, called the degree of freedom of the factor. Generally speaking, the degree of freedom is equal to the independent variable, minus its derivative quantity. The definition of variance is the sum of the square of the sample minus the mean, so for N random samples, the degree of freedom is N-1. For example, the control factor A in this experiment has two levels, so the DOF is one.

### 3.2. Optimization of Process Parameters for Average Temperature as a Single Quality Characteristic

**Figure 9:**
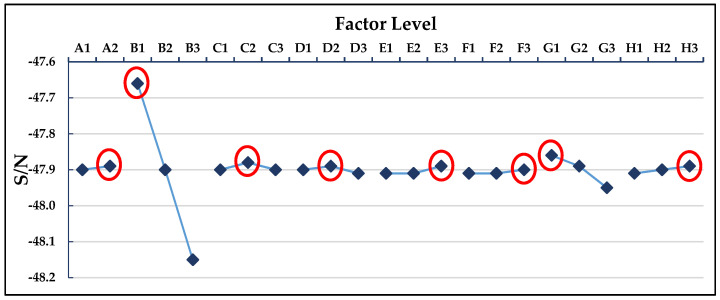
S/N response graph for average temperature.

The *L*_18_ (2^1^ × 3^7^) orthogonal table was used for the experimental simulation of 18 groups of process parameter combinations. This part is the experimental part in (3.1) and (3.2) of Figure 7. The average temperature of each group was simulated and the S/N value of each group was calculated, respectively; the results are shown in Table 9. Table 10 depicts the S/N factor response, and Figure 9 depicts the S/N factor response graph. This part is the experimental calculation result part in (3.3) of Figure 7. The degree of impact of each factor on the average temperature can be known from the factor response table and graph, which are in the following order: cooling liquid, holding pressure, cooling temperature, holding time, mold temperature, filling time, plastic temperature, and injection pressure. The optimized process parameter combination is A2B1C2D2E3F3G1H3, where A2 is the cooling liquid (oil), B1 is the holding pressure (190 MPa), C2 is the cooling temperature (30 °C), D2 is the holding time (1.5 s), E3 is the mold temperature (60 °C), F3 is the filling time (2 s), G1 is the plastic temperature (255 °C), and H3 is the injection pressure (150 MPa). However, this process parameter combination is not found in the *L*_18_ (2^1^ × 3^7^) orthogonal table, suggesting that a confirmation experiment must be conducted to compare with the group comprising the lowest average temperature in the *L*_18_ (2^1^ × 3^7^) orthogonal table, as shown in Table 11. The 12th group has the lowest average temperature among the 18 groups, with an average temperature of 239.57 °C, while the average temperature of the optimized parameter combination is 235.23 °C. Therefore, we can conclude that A2B1C2D2E3F3G1H3 is the optimized process parameter combination with regard to the average temperature. This part is the experimental result part in (3.4) of Figure 7.

In terms of ANOVA, as shown in Table 12, factor B, the holding pressure, will directly affect the plastic temperature in the filling stage if its value is overly high or low. Thus, the holding pressure is the parameter that has the greatest effect on the results of the average temperature among the eight factors, followed by factor G, the plastic temperature; factor A, the cooling liquid; factor E, the mold temperature; factor D, the holding time; factor H, the injection pressure; and factor F, the filling time. Factor C, the cooling temperature, has the smallest contribution, indicating that factor C has the smallest effect on the average temperature results.

## 4. Optimized Process Parameters of Quality Characteristics

### 4.1. Gray Correlation Generation and Gray Correlation Coefficient of Gray Correlation Analysis

In this section, the S/N values that were obtained in the previous section are gray correlated with the S/N values (Table 5 and Table 9) of the 18 groups of parameter combinations for the two single quality characteristics. This part is the experimental calculation result part in (4) of Figure 7. In addition, the S/N values of different intervals are converted to values between zero and one, i.e., the data are normalized, as shown in Table 13. Since a larger S/N value is better, the larger-the-better (LTB) in Equation (2) was used for the calculation. The normalized S/N values for each quality characteristic were then substituted into Equation (3), to calculate the gray correlation coefficient, as shown in Table 14.

### 4.2. Fuzzy Inference System

**Figure 10:**
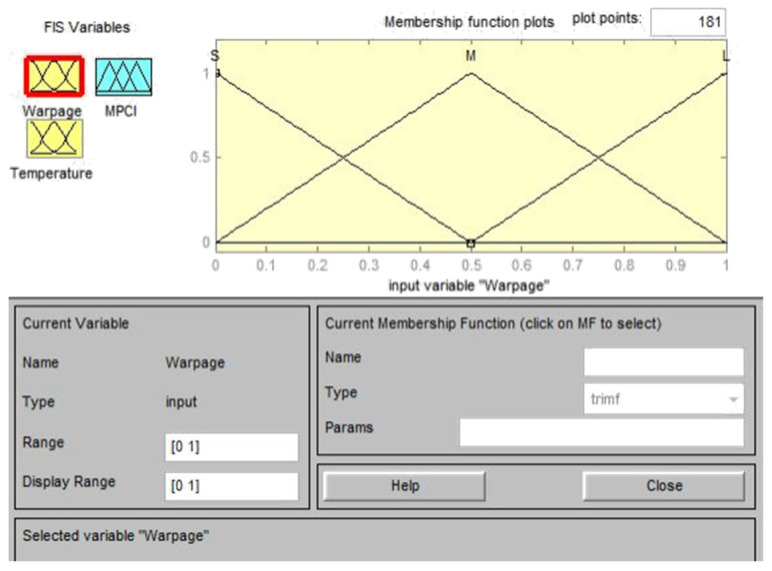
Membership function of input variables.

**Figure 11:**
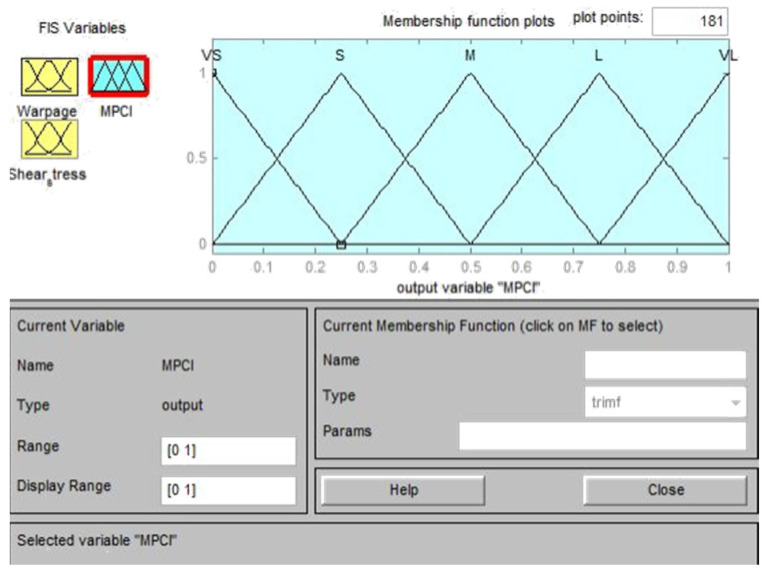
Membership function of the output variable.

**Figure 12:**
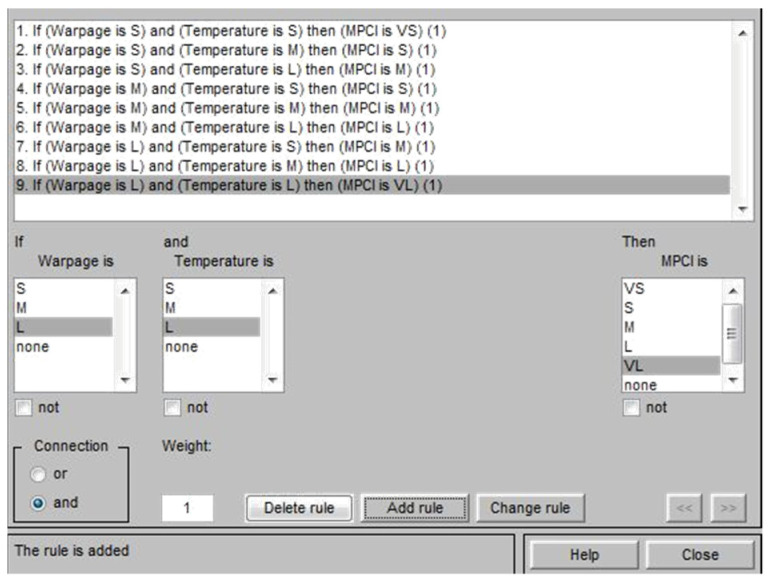
Fuzzy rule base.

**Figure 13:**
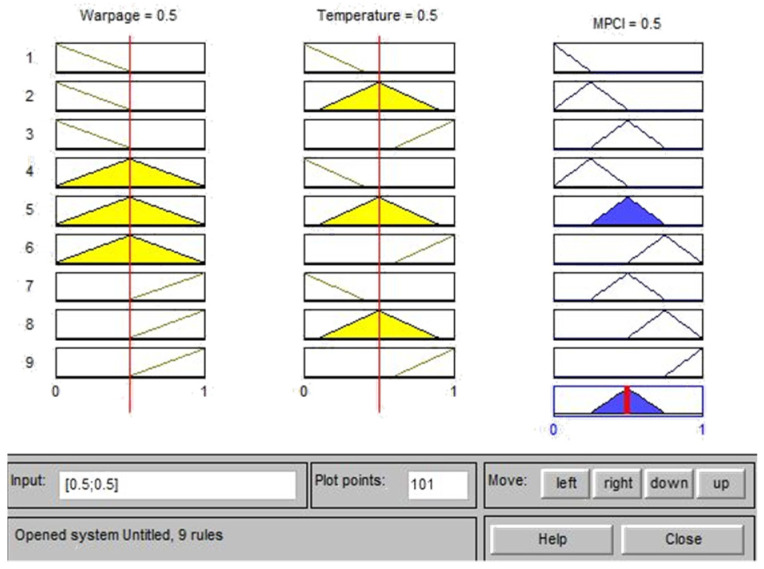
Fuzzy inference process diagram.

In this study, the fuzzy theory method is used to obtain the optimized process parameters of multiple quality characteristics, by considering the following two quality characteristics: the warpage deformation and average temperature. This part is the experimental calculation result part in (5) of Figure 7. This method uses the Matlab fuzzy tool to treat the gray correlation coefficient of the warpage deformation and average temperature as fuzzy rules for the input and output variables of the fuzzy theory method [30,31]. Triangular membership functions were used herein as fuzzy rules of input and output variables, as shown in Figure 10 and Figure 11, respectively. Table 4 was imported into the Matlab fuzzy tool and defined as a fuzzy rule base, as shown in Figure 12. After using Mamdani’s fuzzy inference method for fuzzy inference, the MPCI was obtained through the center of gravity (COG) defuzzification method. This part is the part of the experimental calculation results in (5.1) of Figure 7, as shown in Figure 13. The data input into the fuzzy inference system are in Table 14, after the calculation of the two respective targets is completed, and the gray correlation coefficients of quality characteristics. After the Matlab fuzzy is formulated, the tool defined as a fuzzy rule base passes through the attribution function in the fuzzy inference system, to map a clear value to the fuzzy attribution degree generated by other corresponding attribution function graphs. This process is defuzzification. After defuzzification, the 18 groups of two in Table 15 are used to obtain the MPCI value under the quality characteristics objectives.

**Figure 14:**
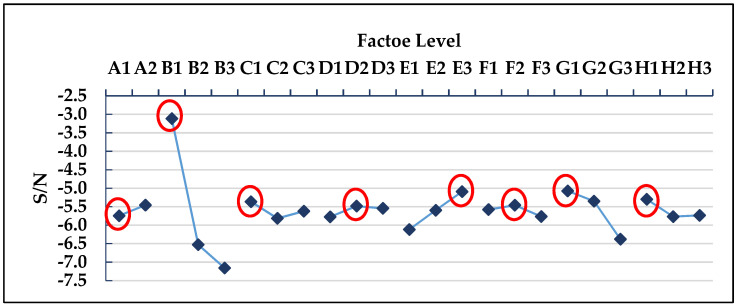
S/N factor response diagram of the measurement indicator (MPCI).

Table 15 presents the MPCI values and S/N ratios that were calculated by the fuzzy system, Table 16 presents the S/N factor response, and Figure 14 depicts the S/N factor response graph. The degree of impact of each factor on the MPCI value can be known from the factor response table and graph, which are in the following order: the holding pressure, plastic temperature, mold temperature, injection pressure, cooling temperature, filling time, holding time, and cooling liquid. The multiple quality optimization process parameter combination is A2B1C1D2E3F2G1H1, where A2 is the cooling liquid (oil), B1 is the holding pressure (190 MPa), C1 is the cooling temperature (15 °C), D2 is the holding time (1.5 s), E3 is the mold temperature (60 °C), F2 is the filling time (1.5 s), G1 is the plastic temperature (255 °C), and H1 is the injection pressure (110 MPa). However, this process parameter combination is not found in the *L*_18_ (2^1^ × 3^7^) orthogonal table, suggesting that a confirmation experiment must be conducted to compare with the group comprising the highest MPCI in the *L*_18_(2^1^ × 3^7^) orthogonal table, as shown in Table 17. The 10th group has the highest MPCI among the 18 groups, with a warpage of 0.807 mm and an average temperature of 240.81 °C, while the warpage and average temperature of the optimized parameter combination are 0.753 mm and 238.71 °C, respectively. Therefore, A2B1C1D2E3F2G1H1 can be concluded to be the optimized process parameter combination with regard to MPCI. This part is the experimental result part in (5.2) of Figure 7.

In terms of ANOVA, as shown in Table 18, factor B, the holding pressure, has the greatest impact on the warpage and average temperature results among the eight factors, followed by factor G, the plastic temperature; factor E, the mold temperature; factor H, the injection pressure; factor C, the cooling temperature; factor A, the coolant; and factor F, the filling time. Factor D, the holding time, has the smallest contribution, indicating that factor D has the smallest effect on the warpage and average temperature results.

According to the experimental results, factor B is the factor with the highest contribution of the single target of warpage deflection and average temperature, and MPCI optimization. The value of the holding pressure for level one is 190 MPa. The pressure value being smaller is better, but it is not suitable for the holding pressure to be too small. Otherwise, it will affect the accuracy of the molded product. The holding pressure is to seal the sprue at the end of injection, and compensate for volume shrinkage. Therefore, the holding pressure must be higher than the internal residual pressure. When the holding pressure or holding time increases, although it can reduce the shrinkage of the product and the surface depression, it will cause excessive pressure near the gate, forming burrs and stress concentration, resulting in serious warp-age and deformation; the pressure applied before the plastic solidifies. The larger the volume, the smaller the volume shrinkage there. The pressure in the mold cavity usually decreases from the gate to the filling end, so the volume shrinkage from the far end of the gate is usually greater than that near the gate. On the contrary, if the holding pressure is too low, it will cause the injection pressure to flow back, causing the molded product to shrink and deform, and affect the precision of the molded product. Another reason for this study is the industry–academic cooperation plan with industry manufacturers. The manufacturers hope to redesign and manufacture molds without increasing operating costs, while maintaining mass production on the existing production lines to save the most costs. For this reason, the method should be adjusted to the process parameters, in order to optimize the quality of the process. In the optimization process, the original process parameters of the manufacturer must be considered. If the holding pressure is too small, it will be necessary to adjust the factor levels of other process parameters, such as the holding time, filling time, etc. The processing time may be lengthened as a whole. Generally, injection molding is carried out in mass production. If the production time of each molded product increases, it may cause a large increase in the production costs and lower the production benefits.

### 4.3. Comparison of Different Cooling Circuit System Designs

**Figure 15:**
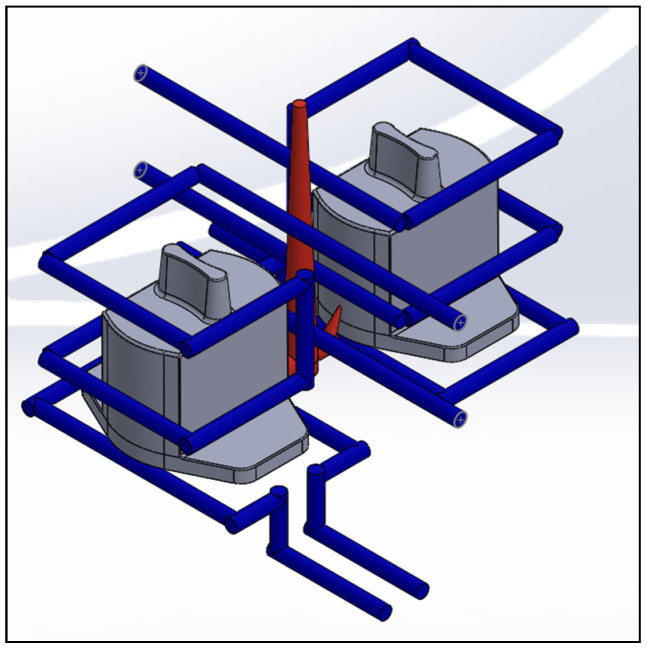
Cooling circuit design of original cooling.

**Figure 16:**
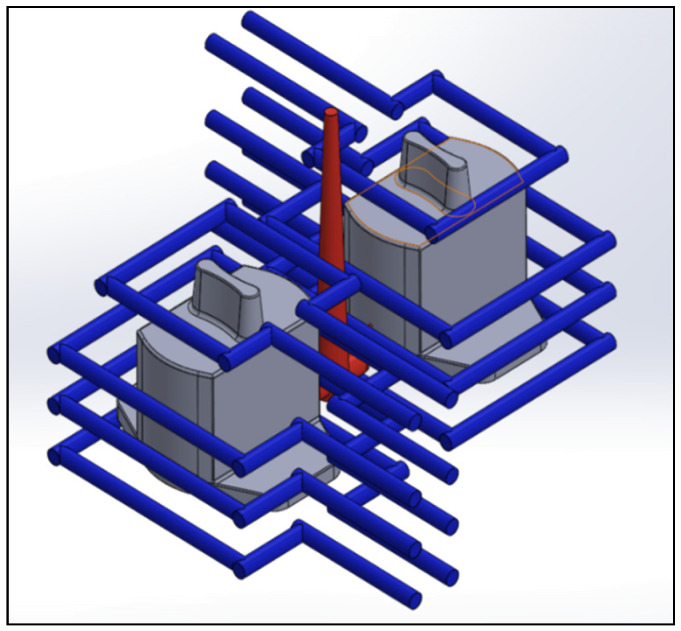
Cooling circuit design of square cooling.

**Figure 17:**
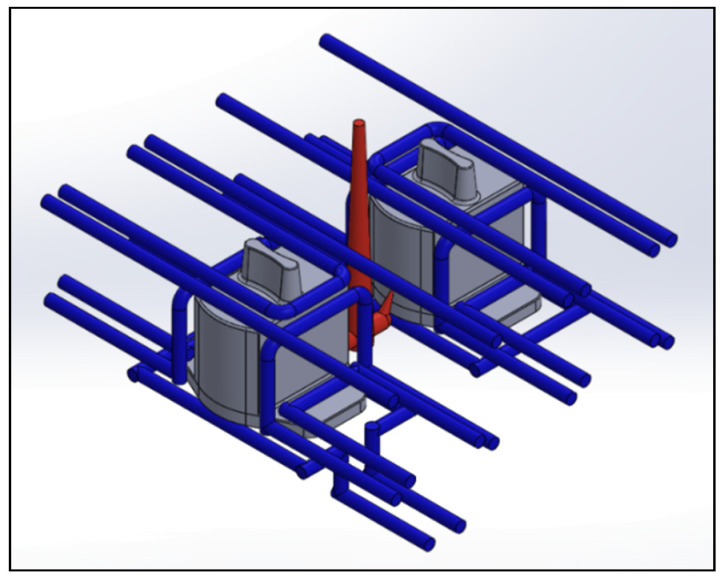
Cooling circuit design of conformal cooling.

Herein, the effects of different cooling circuit systems, based on the optimization process parameters of multi-objective quality characteristics obtained from the above analysis, are discussed and compared. This part is the experimental result part in (6) of Figure 7. At the end of the cooling process of injection molding, if the temperature distribution of the mold is uniform, it means that the cooling circuit is well designed. In contrast, if the temperature distribution of the mold is highly uneven, the plastic part will be easily warped and deformed, due to the thermal stress caused by the difference in temperature. Therefore, if the cooling circuit is not properly designed, the molding time will increase, and the uneven cooling will also cause the plastic part to warp and deform. Three different cooling circuit configurations are discussed in this study, including original cooling, square cooling, and conformal cooling, as shown in Figure 15, Figure 16 and Figure 17. To effectively remove the heat from the mold, square cooling has four groups of cooling circuits surrounding the part, while conformal cooling is designed according to the shape of the auto lock part to wrap around it, and then the cooling circuits are added to the part that cools the slowest, according to an analysis to increase the cooling efficiency. A comparison of the three different cooling circuit designs is shown in Table 19. The analysis will be used to compare whether the conformal cooling circuits improve the auto lock parts, and to understand the effect of the conformal cooling circuits on the average temperature and warpage deformation of the parts.

### 4.4. Effect of Different Cooling Circuit Design Methods on Warpage Deformation

**Figure 18:**
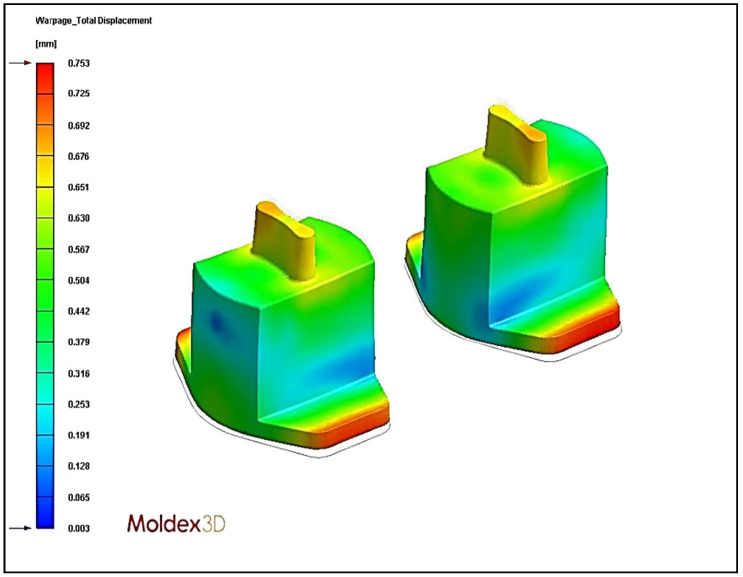
Warpage displacement diagram of original cooling.

**Figure 19:**
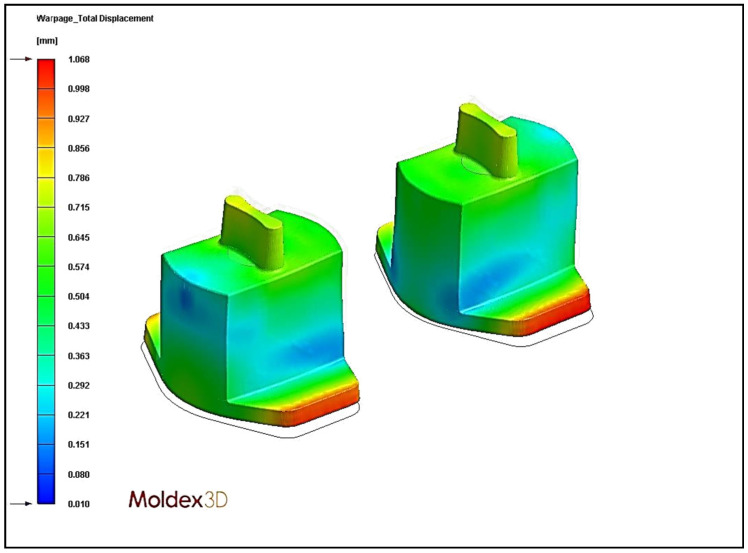
Warpage displacement diagram of square cooling.

**Figure 20:**
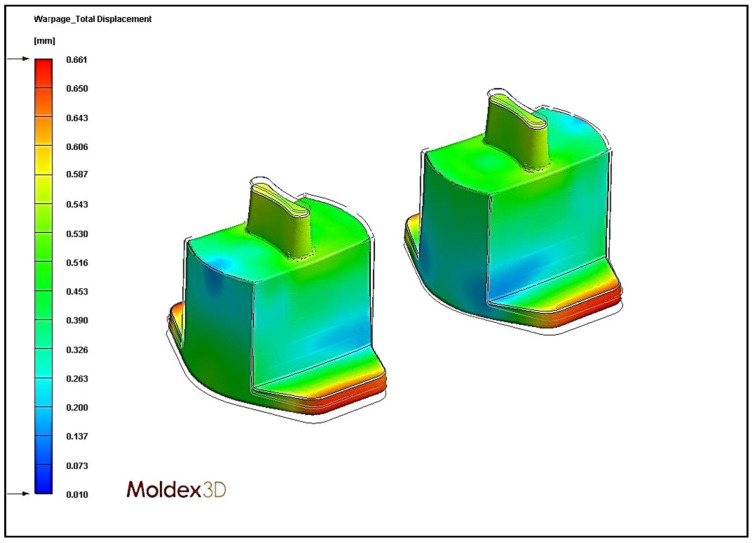
Warpage displacement diagram of conformal cooling.

**Figure 21:**
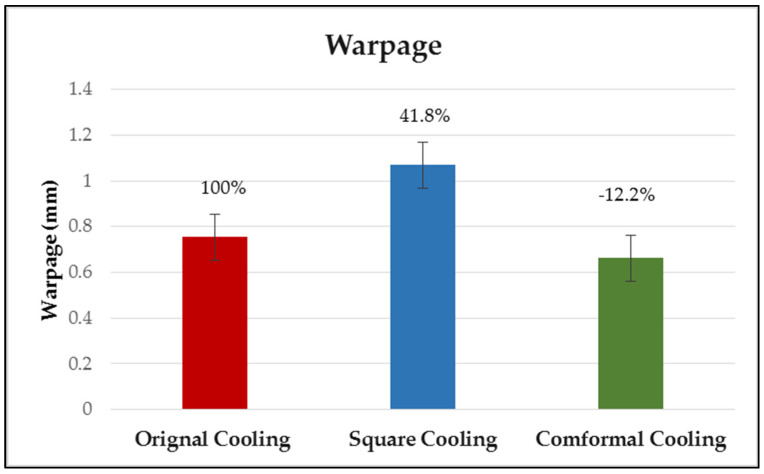
Comparison of warpage deformation values for different cooling circuit systems.

Herein, the effects of three cooling system designs on warpage deformation are discussed. The experimental cooling systems are original cooling, square cooling, and conformal cooling. The warpage deformation displacement diagrams are shown in Figure 18, Figure 19 and Figure 20. According to the results, the conformal cooling design has a better warpage value. As shown in Figure 21, the warpage is 0.661 mm with the conformal channel, 0.753 mm with the original channel, and 1.068 mm with the square channel. In square cooling, compared to original cooling, the warpage value is 41.80% higher. In conformal cooling, the warpage value of original cooling is reduced by 12.20%. In addition to the original horizontal cooling circuit configuration, conformal cooling has a ring-shaped configuration in the vertical direction of the plastic part. Consequently, the plastic parts can be cooled with more complete coating. The overall temperature difference of the plastic parts during cooling can be effectively controlled, and the warpage deformation of the plastic parts will not be affected by the excessive temperature difference.

### 4.5. Effect of Different Cooling Circuit Design Methods on Average Temperature

**Figure 22:**
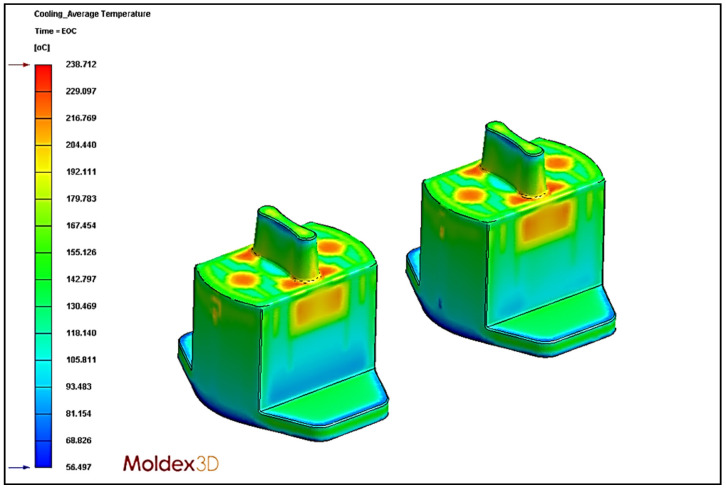
Average temperature diagram of original cooling.

**Figure 23:**
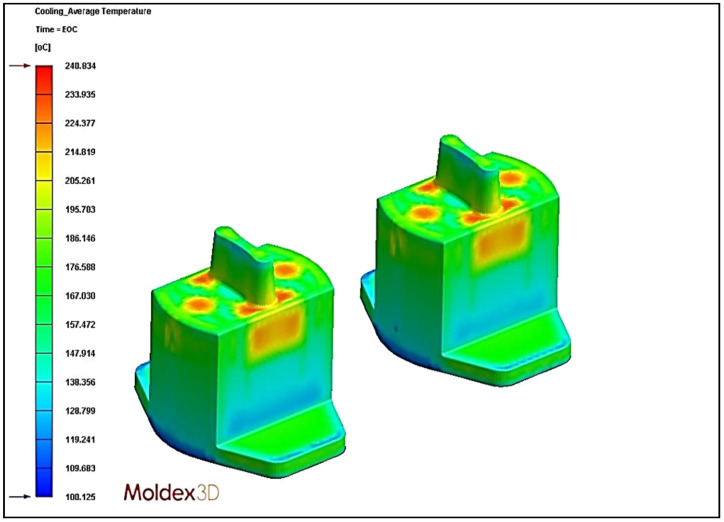
Average temperature diagram of square cooling.

**Figure 24:**
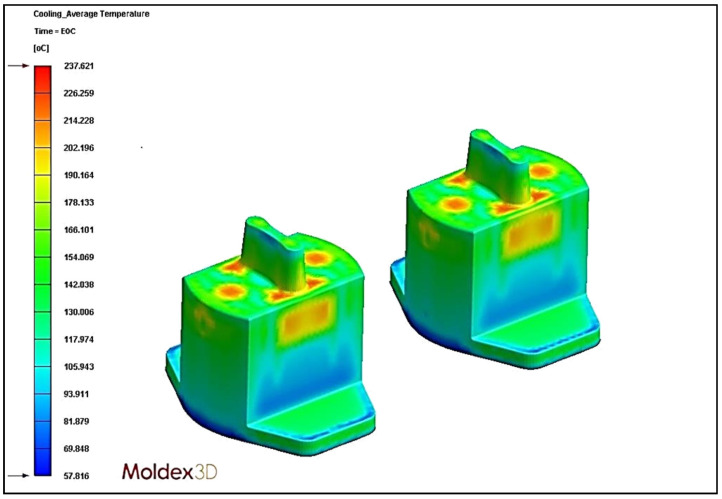
Average temperature diagram of conformal cooling.

**Figure 25:**
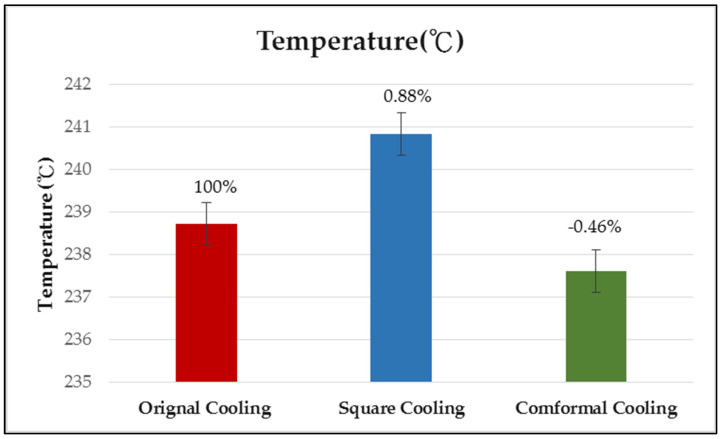
Comparison of average temperature of different cooling circuit systems.

In this section, the effects of three different cooling circuit designs on the average temperature are discussed. The experimental cooling systems are original cooling, square cooling, and conformal cooling. The average temperature diagrams are shown in Figure 22, Figure 23 and Figure 24. According to the results, the conformal cooling design has the lowest average temperature; as shown in Figure 25, the average temperature is 237.62 °C with conformal cooling, 238.71 °C with original cooling, and 240.83 °C with square cooling. In square cooling, the average temperature is 0.88% higher than that of original cooling. In conformal cooling, the average temperature of original cooling is reduced by 0.46%. The analysis results show that the average temperature with square cooling is higher than that with original cooling. The reason for this may be that the layers are too densely stacked in the design of square cooling, which causes the high temperatures between the cooling circuits to affect each other, resulting in poor heat dissipation. In conformal cooling, on the other hand, the cooling circuits are closely attached to the plastic parts; thus, they can cool and dissipate heat in multiple axes to obtain the optimal average temperature value.

## 5. Conclusions

The Taguchi method is a reliable design method and concept of statistical experiment design. It streamlines the number of experiments and improves the method of analyzing experimental data, thereby stabilizing the quality while reducing the manufacturing costs. Using the orthogonal table OA, many design variables can be analyzed in a small amount, which can greatly reduce the number of experiments and improve the accuracy of the experiment. The fuzzy logic program used in MPCI can reduce the uncertainty caused by artificiality, and this method does not require complicated mathematical calculations. In this system simulation study, fuzzy logic is applied in MPCI. Compared with the traditional method, the output can meet the requirements of technical engineers and customers more comprehensively.

The experimental results are summarized and organized as follows:

1. The combination of parameters that optimizes the warpage deformation of auto lock parts can be obtained from the experimental results of the Taguchi robust design in combination with optimization analysis, and the injection molding conditions are A2B1C1D3E3F2G1H1, where A2 is the cooling liquid (oil), B1 is the holding pressure (190 MPa), C1 is the cooling temperature (15 °C), D3 is the holding time (2 s), E3 is the mold temperature (60 °C), F2 is the filling time (1.5 s), G1 is the plastic temperature (255 °C), and H1 is the injection pressure (110 MPa). The best warpage deformation of the original orthogonal is 0.807 mm, whereas that for the optimized parameter combination is 0.783 mm. After optimization, the warpage deformation can be increased by 2.97% compared to the optimal group in the original orthogonal table;
2. The combination of parameters that optimizes the average temperature of auto lock parts can be obtained from the experimental results of the Taguchi robust design in combination with optimization analysis, and the injection molding conditions are A2B1C2D2E3F3G1H3, where A2 is the cooling liquid (oil), B1 is the holding pressure (190 MPa), C2 is the cooling temperature (30 °C), D2 is the holding time (1.5 s), E3 is the mold temperature (60 °C), F3 is the filling time (2 s), G1 is the plastic temperature (255 °C), and H3 is the injection pressure (150 MPa). The best average temperature of the original orthogonal is 239.57 °C, while that of the optimized parameter combination is 235.23 °C. After optimization, the average temperature can be increased by 1.70% compared to the optimal group in the original orthogonal table;
3. Fuzzy theory analysis is used to obtain a set of optimal process parameters for multi-objective quality characteristics, which are warpage deformation and average temperature, and the experimental results show that the optimal process parameters for multi-objective quality characteristics are A2B1C1D2E3F2G1H1. Here, A2 is the cooling liquid (oil), B1 is the holding pressure (190 MPa), C1 is the cooling temperature (15 °C), D2 is the holding time (1.5 s), E3 is the mold temperature (60 °C), F2 is the filling time (1.5 s), G1 is the plastic temperature (255 °C), and H1 is the injection pressure (110 MPa). As the best group in the original orthogonal array, warpage deformation on a single target is 0.807 mm, and the average temperature is 239.57 °C. Those that were optimized for the multi-objective parameter combination are 0.753 mm and 238.71 °C, respectively. When both the objectives were considered, both quality characteristics will increase by 7.17% in warping deformation, and 0.3% in average temperature;
4. Analysis of the effect of different cooling circuit systems shows the difference between the traditional channel and the conformal channel. The results show that the warpage deformation with conformal cooling is smaller than that with original cooling. Moreover, conformal cooling improves the average temperature. The overall warpage difference is 0.092 mm, and the average temperature difference is 1.091 °C. Furthermore, it is smaller than the original cooling system; it reduces warpage by 12.2% and the average temperature by 0.46%;
5. The system simulation in this study applied fuzzy logic in MPCI. Compared with conventional methods, the output can better meet the requirements of technical engineers and customers. Practical applications can further encourage the transfer of fuzzy logic-based technology from academia to industry. The research results of the optimization modeling method that was proposed by this study show that the injection molding process can be optimized for multiple single goals, and can also be optimized to consider multiple goals and multi-quality characteristics. Moreover, it can greatly reduce the number and time of the experiments, and improve the accuracy of the experiments.

## Figures and Tables

**Table 1:** PA66 (6212GC) material characteristics.

Mechanical Properties	PA66
Density	1.14 (g/cc)
Poisson’s ratio	0.3
Modulus E	$2\times 10^{10}$ (dyne/cm^2^)
CLTE	$7.5\times 10^{-5}$ (1/K)
Fiber Weight Percentage	33 (%)
Melt Temperature	275–305 (°C)

**Table 2:** Comparison of actual and simulated warpage value measurements.

Point	Actual Measurement (mm)	Simulation (mm)	Error (%)
1	0.646	0.649	1.00
2	0.641	0.650	1.01
3	0.521	0.550	1.06
4	0.635	0.706	1.03
5	0.635	0.640	1.01
6	0.538	0.550	1.02
7	0.232	0.226	0.97
8	0.235	0.228	0.97
9	0.174	0.175	1.01
10	0.158	0.157	0.99
11	0.433	0.437	1.01
12	0.411	0.423	1.03
AVG	0.438	0.449	1.03

**Table 3:** Control factors and levels.

Control Factors	Level		
	1	2	3
A. Cooling Liquid	Oil	Water	
B. Holding Pressure (MPa)	190	200	210
C. Cooling Temperature (°C)	15	30	45
D. Holding Time (s).	1	1.5	2
E. Mold Temperature.(°C)	20	40	60
F. Filling Time (s)	1	1.5	2
G. Plastic Temperature (°C)	255	265	275
H. Injection Pressure (s)	110	130	150

**Table 4:** Fuzzy rules with two inputs and one output.

No.	Input		Output
	Warpage	Average Temperature	(MPCI)
1	S	S	VS
2	S	M	S
3	S	L	M
4	M	S	S
5	M	M	M
6	M	L	L
7	L	S	M
8	L	M	L
9	L	L	VL

Input: small (S), middle (M), large (L). Output: very small (VS), small (S), middle (M), large (L), very large (VL).

**Table 5:** S/N values of warpage deflection groups.

No.	Warpage (mm)	S/N
1	0.961	0.341
2	0.948	0.553
3	0.945	0.581
4	1.092	−0.761
5	0.944	0.496
6	1.051	−0.434
7	1.108	−0.887
8	1.140	−1.135
9	0.993	0.059
10	0.807	1.861
11	1.011	−0.098
12	0.857	1.341
13	1.036	−0.309
14	1.043	−0.367
15	1.013	−0.116
16	1.032	−0.271
17	1.050	−0.427
18	1.204	−1.612

**Table 6:** S/N response table of warpage deflection.

Factor	A	B	C	D	E	F	G	H
Level 1	−0.13	0.76	−0.004	−0.17	−0.51	−0.10	0.27	−0.03
Level 2	0	−0.25	−0.16	−0.07	−0.07	−0.04	0.09	−0.08
Level 3		−0.71	−0.03	0.04	0.38	−0.05	−0.56	−0.09
Effect	0.13	1.48	0.16	0.21	0.89	0.06	0.82	0.06
Rank	6	1	5	4	2	7	3	8
Optimal parameters	A2	B1	C1	D3	E3	F2	G1	H1

**Table 7:** Confirmation test of warpage deflection.

No.	Factor	Warpage (mm)	S/N (dB)
Original (10)	A2B1C1D3E3F2G2H1	0.807	1.861
Optimization	A2B1C1D3E3F2G1H1	0.783	2.122

**Table 8:** Variance analysis of warpage deformation.

Factor	DOF	Seq SS	F	P	Confidence	Contribution
A	1	0.08	0.41	0.59	41.2 (%)	0.65 (%)
B	2	6.83	17.74	0.05	94.7 (%)	56.25 (%)
C	2	0.09	0.23	0.82	18.4 (%)	0.72 (%)
D	2	0.13	0.33	0.75	24.9 (%)	1.05 (%)
E	2	2.36	6.12	0.14	86 (%)	19.41 (%)
F	2	0.01	0.03	0.97	3.3 (%)	0.11 (%)
G	2	2.25	5.85	0.15	85.4 (%)	18.54 (%)
H	2	0.01	0.03	0.97	3.3 (%)	0.11 (%)
Error	2	0.39				3.16 (%)
Total	17	12.14				100 (%)

**Table 9:** S/N values for average temperature groups.

No.	Average Temperature (°C)	S/N
1	241.15	−47.655
2	241.88	−47.670
3	242.79	−47.705
4	250.24	−47.967
5	247.17	−47.860
6	249.20	−47.931
7	254.78	−48.123
8	257.91	−48.229
9	254.24	−48.105
10	240.81	−47.634
11	242.71	−47.702
12	239.57	−47.589
13	248.86	−47.919
14	247.09	−47.857
15	247.83	−47.883
16	254.05	−48.099
17	254.83	−48.125
18	257.10	−48.202

**Table 10:** S/N response table for average temperature.

Factor	A	B	C	D	E	F	G	H
Level 1	−47.90	−47.66	−47.90	−47.90	−47.91	−47.91	−47.86	−47.91
Level 2	−47.89	−47.90	−47.88	−47.89	−47.91	−47.91	−47.89	−47.90
Level 3		−48.15	−47.90	−47.91	−47.89	−47.90	−47.95	−47.89
Effect	0.03	0.49	0.01	0.02	0.02	0.01	0.09	0.01
Rank	3	1	8	5	4	7	2	6
Optimal parameters	A2	B1	C2	D2	E3	F3	G1	H3

**Table 11:** Confirmation experiment for average temperature.

No.	Factor	Average Temperature (°C)	S/N (dB)
Original (12)	A2B1C3D2E2F1G1H3	239.57	−47.589
Optimization	A2B1C2D2E3F3G1H3	235.23	−47.430

**Table 12:** Variance analysis for average temperature.

Factor	DOF	Seq SS	F	P	Confidence	Contribution
A	1	0.00283	12.10	0.074	92.60 (%)	0.38 (%)
B	2	0.71986	1526.4	0.001	99.90 (%)	95.46 (%)
C	2	0.00026	0.55	0.647	35.30 (%)	0.03 (%)
D	2	0.00075	1.59	0.386	61.40 (%)	0.10 (%)
E	2	0.00119	2.52	0.284	71.60 (%)	0.16 (%)
F	2	0.00048	1.01	0.498	50.20 (%)	0.06 (%)
G	2	0.02757	58.46	0.017	98.30 (%)	3.66 (%)
H	2	0.00067	1.41	0.414	58.60 (%)	0.09 (%)
Error	2	0.00047				0.06 (%)
Total	17	0.75408				100 (%)

**Table 13:** Normalized S/N ratio for each quality characteristic.

No.	Warpage S/N Ratio	Average Temperature S/N Ratio
1	0.562	0.897
2	0.623	0.873
3	0.632	0.819
4	0.245	0.409
5	0.607	0.577
6	0.339	0.466
7	0.209	0.166
8	0.137	0
9	0.481	0.194
10	1.000	0.930
11	0.436	0.823
12	0.850	1.000
13	0.375	0.484
14	0.359	0.581
15	0.431	0.541
16	0.386	0.203
17	0.341	0.163
18	0	0.042

**Table 14:** Gray correlation coefficients of quality characteristics.

No.	Warpage (mm)	Average Temperature (°C)
1	0.533	0.829
2	0.570	0.798
3	0.576	0.734
4	0.398	0.459
5	0.560	0.542
6	0.431	0.483
7	0.387	0.375
8	0.367	0.333
9	0.491	0.383
10	1.000	0.877
11	0.470	0.739
12	0.770	1.000
13	0.445	0.492
14	0.438	0.544
15	0.468	0.521
16	0.449	0.386
17	0.432	0.374
18	0.333	0.343

**Table 15:** MPCI values and corresponding S/N ratios.

No.	MPCI	S/N
1	0.688	−3.248
2	0.667	−3.517
3	0.618	−4.180
4	0.439	−7.151
5	0.539	−5.368
6	0.456	−6.821
7	0.429	−7.351
8	0.410	−7.744
9	0.483	−6.321
10	0.882	−1.091
11	0.589	−4.598
12	0.788	−2.069
13	0.464	−6.670
14	0.460	−6.745
15	0.478	−6.411
16	0.463	−6.688
17	0.451	−6.916
18	0.401	−7.937

**Table 16:** S/N response table of the measurement indicator (MPCI).

Factor	A	B	C	D	E	F	G	H
Level 1	−5.745	−3.117	−5.366	−5.774	−6.117	−5.578	−5.073	−5.3
Level 2	−5.458	−6.528	−5.815	−5.485	−5.597	−5.46	−5.351	−5.769
Level 3		−7.16	−5.623	−5.545	−5.091	−5.766	−6.38	−5.735
Effect	0.286	4.042	0.448	0.289	1.026	0.306	1.307	0.469
Rank	8	1	5	7	3	6	2	4
Optimal parameters	A2	B1	C1	D2	E3	F2	G1	H1

**Table 17:** Confirmation experiment of the measurement indicator (MPCI).

No.	Factor	Warpage (mm)	Temperature (°C)
Original (10)	A2B1C1D3E3F2G2H1	0.807	240.81
Optimization	A2B1C1D2E3F2G1H1	0.753	238.71

**Table 18:** Variance analysis of the measurement indicator (MPCI).

Factor	DOF	Seq SS	F	P	Confidence	Contribution
A	1	0.3687	0.54	0.539	46.10 (%)	0.53 (%)
B	2	56.7416	41.50	0.024	97.60 (%)	81.86 (%)
C	2	0.6076	0.44	0.692	30.80 (%)	0.88 (%)
D	2	0.2791	0.20	0.830	17.00 (%)	0.40 (%)
E	2	3.1553	2.31	0.302	69.80 (%)	4.55 (%)
F	2	0.2855	0.21	0.827	17.30 (%)	0.41 (%)
G	2	5.6852	4.16	0.194	80.60 (%)	8.20 (%)
H	2	0.8217	0.60	0.625	37.50 (%)	1.19 (%)
Error	2	1.3674				1.97 (%)
Total	17	69.3121				100 (%)

**Table 19:** Comparison of three different cooling circuit systems.

Cooling Circuit Type	Number of Inlets	Number of Outlets	Reynolds Number of a Single Pipe	Cooling Liquid	Inlet Oil Temperature
Original Cooling	2	2	6570	Oil	30 ℃
Square Cooling	6	6	6570	Oil	30 ℃
Conformal Cooling	11	11	6570	Oil	30 ℃

## Data Availability

The authors collected the data by themselves using the proposed method for this article.
